# Development of an indirect ELISA based on a multi-epitope recombinant protein for antibody detection against African swine fever virus

**DOI:** 10.1128/spectrum.03525-25

**Published:** 2026-05-21

**Authors:** Lixinjie Liu, Qichao Chen, Linmeng Yu, Wei Wu, Shuang Mou, Huihui Guo, Chen Tan, Qingyun Liu, Dang Wang, Huanchun Chen, Xiangru Wang

**Affiliations:** 1 National Key Laboratory of Agricultural Microbiology, College of Veterinary, Huazhong Agricultural University 47895, Wuhan, Hubei, China; 2 Key Laboratory of Preventive Veterinary Medicine in Hubei Province, The Cooperative Innovation Center for Sustainable Pig Production, Wuhan, China; 3 Engineering Research Center of Animal Biopharmaceuticals, The Ministry of Education of the People’s Republic of China (MOE), Wuhan, China; 4 Key Laboratory of Prevention & Control for African Swine Fever and Other Major Pig Diseases, Ministry of Agriculture and Rural Affairs, Wuhan, China; Changchun Veterinary Research Institute, Chinese Academy of Agricultural Sciences, Changchun, China

**Keywords:** African swine fever virus, multi-epitope antigen, indirect ELISA

## Abstract

African swine fever (ASF) is a highly contagious and fatal hemorrhagic disease of swine. While vaccines have been licensed in Vietnam and the Philippines, their global application remains constrained by insufficient large-scale validation of safety and efficacy, rendering rapid and reliable diagnostic assays crucial for effective prevention and control. In this study, a multi-epitope antigen (MEA) was rationally designed by utilizing informatics to predict and select 15 conserved B-cell epitopes from four major African swine fever virus (ASFV) structural proteins: p30, p54, p72, and pA104R. The recombinant MEA was expressed in *Escherichia coli,* and its immunogenicity was validated by Western blot and indirect immunofluorescence assays. This antigen was subsequently utilized as the basis for an indirect enzyme-linked immunosorbent assay (iELISA) whose reaction conditions were systematically optimized. The optimal cutoff value for the iELISA was determined to be 0.315 from the analysis of 100 known ASFV-negative serum samples. The assay demonstrated excellent reproducibility, with intra- and inter-assay coefficients of variation below 10%, and exhibited high specificity, with no cross-reactivity against sera positive for other common swine viruses. Notably, the developed assay exhibited high sensitivity, capable of detecting ASFV antibodies in serum samples at dilutions up to 1:6,400. Furthermore, it demonstrated superior sensitivity compared to two commercial ELISA kits in validating clinical samples from regions with circulating Genotype II strains. These findings highlight the successful development of a sensitive and specific iELISA for ASFV antibody detection, representing a valuable new tool for large-scale epidemiological surveillance and the implementation of robust control strategies for ASF.

**IMPORTANCE:**

African swine fever (ASF) is a devastating viral disease of swine causing substantial economic losses globally. Although vaccines have recently been licensed in countries such as Vietnam and the Philippines, the global availability of a universally standardized and safe vaccine remains a significant challenge. Control efforts continue to rely heavily on rapid and accurate diagnosis. A significant limitation of current serological assays is their variable sensitivity, which can lead to missed detections and compromise surveillance programs. This study addresses this deficiency through a rational, epitope-based engineering approach. We designed a single, chimeric antigen by genetically fusing 15 conserved, immunodominant B-cell epitopes from four major African swine fever virus proteins. The resulting indirect enzyme-linked immunosorbent assay demonstrated high specificity, excellent reproducibility, and, critically, superior diagnostic sensitivity compared to two commercial kits. This work reports the development of a significantly improved serodiagnostic tool, offering enhanced reliability for large-scale epidemiological surveillance and supporting more robust control strategies against ASF.

## INTRODUCTION

African swine fever (ASF) is a devastating, highly contagious viral disease that affects both domestic pigs and wild boar. The rapid global spread of the causative agent, African swine fever virus (ASFV), is a complex, enveloped DNA virus that possesses a large genome encoding over 150 proteins. This genomic complexity, coupled with significant immunological diversity, poses a formidable challenge to ASF control. Sequence comparisons indicate that key structural proteins share sequence similarities ranging from approximately 84% to 97.9% among different strains, which complicates the development of universally applicable vaccines and diagnostic assays. Although ASFV vaccines were licensed in limited regions, widespread global adoption remains a challenge. Consequently, the control and eradication of ASF remain heavily reliant on early, rapid, and accurate diagnosis, followed by the implementation of strict quarantine and culling measures.

Serological assays for antibody detection are indispensable components of ASF surveillance programs, serving to identify subacute or chronic infections and detect infections caused by low-virulence strains (1, 2). The World Organization for Animal Health (WOAH) has designated the enzyme-linked immunosorbent assay (ELISA) as a standard method for the serological diagnosis of ASFV infection (3). While several commercial ELISA kits are available worldwide, including the competitive ELISA INGEZIM PPA COMPAC (INGENASA, Spain) and indirect ELISAs (iELISAs) such as ID Screen African Swine Fever Indirect (IDvet, France) and SVANOVIR ASFV-Ab (Svanovir, Sweden), their diagnostic performance, particularly in terms of sensitivity and specificity, can vary. This variability is often influenced by factors such as the assay format, the specific viral antigens employed, and the genetic variability of the target antigens (4).

Several ASFV proteins are well recognized for their strong immunogenicity and are frequently employed as diagnostic antigens. The early structural protein p30, encoded by the *CP204L* gene, elicits a robust and early antibody response, rendering it a key target for early serological diagnosis (5, 6). Similarly, the p54 protein, another structural component encoded by the *E183L* gene, induces a strong and persistent antibody response detectable from approximately 8 days post-infection (7, 8). ELISA-based antibody detection methods targeting the p54 protein have been shown to exhibit high sensitivity, specificity, and stability (7). The major capsid protein p72, encoded by the *B646L* gene, is a highly conserved and immunodominant antigen expressed during the late stage of infection and serves as a cornerstone of many serological assays (9–11). Notably, the WOAH-certified reference kit for ASFV diagnosis, the INGENASA competitive ELISA, utilizes a specific monoclonal antibody against the p72 protein to detect viral antigens and demonstrates excellent specificity and sensitivity (3). Additionally, the pA104R protein, expressed during the intermediate to late stages of ASFV infection, has been identified as a highly conserved and antigenic protein that reacts strongly with sera from infected animals (12, 13). Collectively, these antigenic proteins are critical targets for the development of serologic diagnostic tools, thereby aiding in the control and eradication of ASF.

While single-antigen-based ELISAs are widely used, their sensitivity can be limited, leading to false-negative results (14, 15). To address this deficiency, multi-protein strategies have been explored, as they can significantly enhance detection rates by capturing a broader range of antibody responses (16, 17). However, the simple co-presentation of multiple full-length proteins is hindered by several practical and technical challenges, such as high expression costs, batch-to-batch variability in protein quality, and difficulties in optimizing the stoichiometric ratios of the antigens (11). Consequently, the focus has shifted toward epitope-based engineering, as antigenic epitopes represent the fundamental units of immunodominance and can serve as highly specific and sensitive targets for assay development (15, 18). A more advanced approach is the design of a single, chimeric tandem protein constructed from multiple B-cell epitopes derived from several key antigens. This approach has shown significant improvements in sensitivity, enhancing the recognition of diverse antibody populations in swine and reducing the likelihood of inaccurate results when benchmarked against commercial kits (19–21). Such a strategy not only enhances the potential for sensitivity and specificity but also facilitates a more consistent and cost-effective production process. The integration of immunoinformatics for epitope prediction is instrumental in this process, enabling the rational design of antigens with predictably superior diagnostic performance for widespread application (21, 22).

Therefore, this study was undertaken to employ an immunoinformatics approach to identify conserved and immunodominant B-cell epitopes from the p30, p54, p72, and pA104R proteins of ASFV. These selected epitopes were genetically fused to construct a novel multi-epitope antigen (MEA). We describe the expression and purification of this recombinant protein, as well as the subsequent development and validation of a highly sensitive and specific indirect ELISA (iELISA) for the detection of ASFV antibodies. Finally, the diagnostic performance of this MEA-iELISA was benchmarked against existing commercial kits, highlighting its potential as a superior tool for ASF surveillance and control.

## RESULTS

### Design and *in silico* analysis of the multi-epitope antigen

**Fig 1:**
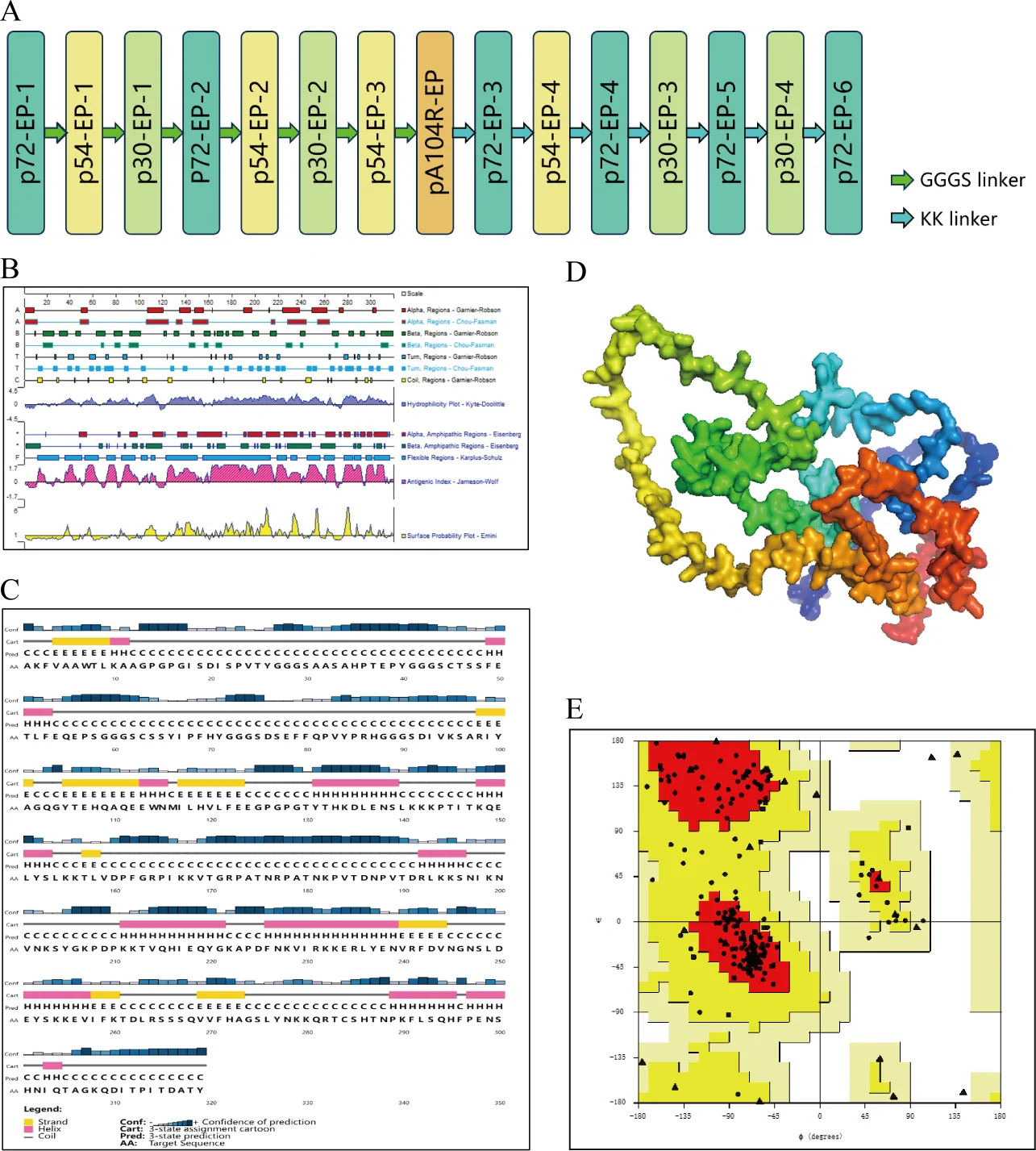
Design and *in silico* characterization of the multi-epitope antigen. (**A**) Schematic representation of the overall architecture of the MEA. (**B**) Analysis of physicochemical properties of the designed MEA. (**C**) A graphical overview of the secondary structural elements of the constructed multi-epitope protein. (**D**) The predicted three-dimensional structure of the MEA. (**E**) The Ramachandran plot assessing the stereochemical quality of the protein’s tertiary structure.

To construct a novel diagnostic antigen, immunoinformatic tools (Immune Epitope Database [IEDB] and SVMTriP) were employed to predict B-cell epitopes from the structural proteins p30, p54, and p72 of the Genotype II strain ASFV SY18 (Table 1). A total of 15 high-scoring epitopes were selected based on their predicted antigenicity and a lack of toxicity or allergenicity. The final construct, termed MEA, was composed of four epitopes from p30, four from p54, six from p72, and one previously identified epitope from pA104R (Table 2). These epitopes were genetically fused using a combination of flexible “GGGS” and rigid “KK” linkers to minimize steric hindrance and optimal surface exposure of each antigenic determinant (Fig. 1A).

**TABLE 1:** Epitope prediction of ASFV p30, p54, and p72 proteins

Protein	IEDB			SVMTriP		
	Position	Sequence	Length	Position	Sequence	Length
p30	14–24	IFKTDLRSSSQ	11	88–107	HVLFEEETESSASSESIHEK	20
	31–35	SLYNW	5	175–194	LKEEEKEVVRLMVIKLLKKK	20
	68–84	IYAGQGYTEHQAQEEWN	17	15–34	FKTDLRSSSQVVFHAGSLYN	20
	92–114	EEETESSASSESIHEKNDNETNE	23	57–76	TVKYDIVKSAHIYAGQGYTE	20
	122–136	LFEQEPSSEEPKDSK	15			
	146–162	QHIEQYGKAPDFNKVIR	17			
	164–182	HNFIQTIHGTPLKEEEKEV	19			
p54	10–13	YPRH	4	52–71	FSSRKKKAAAAIEEEDIQFI	20
	18–26	LSPVTPPSF	9	140–159	AASAHPTEPYTTVTTQNTAS	20
	56–65	KKKAAAAIEE	10	108–127	ATNRPATNKPVTDNPVTDRL	20
	73–124	PYQDQQWAEVTPQPGTSKPAGATTASAGKPVTGRPATNRPATNKPVTDNPVT	52			
	131–161	TGGPAAAPAAASAHPTEPYTTVTTQNTASQT	31			
	173–178	TYTHKD	6			
p72	31–52	SNIKNVNKSYGKPDPEPTLSQI	22	581–600	MITFALKPREEYQPSGHINV	20
	65–90	KPYVPVGFEYNKVRPHTGTPTLGNKL	26	370–389	EFPGLFIRQSRFIPGRPSRR	20
	116–151	SWQDAPIQGTAQMGAHGQLQTFPRNGYDWDNQTPLE	36	185–204	ERLYENVRFDVNGNSLDEYS	20
	160–171	PFGRPIVPGTKN	12	14–33	GKADKIILAQDLLNSRISNI	20
	193–206	FDVNGNSLDEYSSD	14	627–646	ASAINFLLLQNGSAVLRYST	20
	218–236	GDKMTGYKHLVGQEVSVEG	19	207–226	VTTLVRKFCIPGDKMTGYKH	20
	248–308	LHKPHQSKPILTDENDTQRTCSHTNPKFLSQHFPENSHNIQTAGKQDITPITDATYLDIRR	61			
	311–323	HYSCNGPQTPKYY	13			
	364–370	QKDLVNE	7			
	379–393	SRFIPGRPSRRNIRF	15			
	443–457	VTHTNNNHHDEKLMS	15			
	473–489	TWNISDQNPHQHRDWHK	17			
	499–527	QPTHHAEISFQDRDTALPDACSSISDISP	29			
	563–574	PFHYGGNAIKTP	12			
	589–601	REEYQPSGHINVS	13			

**TABLE 2:** Epitope sequences selected in p30, p54, p72, and pA104R proteins

Protein	Start-end position	Sequence
p30	12–34	EVIFKTDLRSSSQVVFHAGSLYN
	144–162	TVQHIEQYGKAPDFNKVIR
	61–93	DIVKSAHIYACQCYTEHQAQEEWNMILHVLFEE
	115–128	TSSFETLFEQEPS
p54	173–183	TYTHKDLENSL
	103–127	VTGRPATNRPATNKPVTDNPVTDRL
	2–13	DSEFFQPVYPRH
	140–149	AASAHPTEPY
p72	156–165	TLVDPFGRPI
	265–303	QRTCSHTNPKFLSQHFPENSHNIQTAGKQDITPITDATY
	31–45	SNIKNVNKSYGKPDP
	185–204	ERLYENVRFDVNGNSLDEYS
	522–530	ISDISPVTY
	558–566	CSSYIPFHY
pA104R	6–17	KPTITKQELYSL

The resulting MEA construct was then subjected to comprehensive *in silico* characterization. Both the ANTIGENpro and VaxiJen v.2.0 servers predicted the MEA to be highly immunogenic. Concurrently, AllerTop and AllergenFP servers indicated that the protein was non-allergenic. Analysis of its physicochemical properties using the ProtParam server predicted a theoretical isoelectric point (pI) of 9.69 and an instability index of 33.55, the latter of which classifies the MEA as a stable protein. Furthermore, a predicted solubility score of 0.601 suggested that the MEA is likely to be expressed as a soluble protein (Table 3).

**TABLE 3:** The antigenicity, allergenicity, and physical properties of MEA

Properties	Tool	Result
Antigenicity prediction	ANTIGENPro	Predicted Probability of Antigenicity: 0.94
	VaxiJen v.2.0	0.4302 (Probable ANTIGEN)
Allergenicity prediction	AllerTop	PROBABLE NON-ALLERGEN
	AllergenFP	PROBABLE NON-ALLERGEN
Physiochemical properties by ProtParam	Molecular weight	35,161.3
	PI	9.690
	Estimated half-life	4.4 h (mammalian reticulocytes, *in vitro*), >20 h (yeast, *in vivo*), >10 h (*Escherichia coli*, *in vivo*)
	Aliphatic index	57.46
	Instability	33.55 (Stable)
	Grand average of hydropathicity	−0.799
Solubility	Protein-sol	0.601 (Soluble)

Structural analysis using the DNASTAR Protean program revealed that the MEA possesses a high antigenic index and surface probability, suggesting strong potential for antibody binding (Fig. 1B). The predicted secondary structure of the MEA, as determined by the PSIPRED v.4.0 server, was composed of approximately 23% alpha-helices, 7% beta-sheets, and 52% random coils (Fig. 1C). This predicted structure, comprising both stable elements and flexible random coils, is conducive to maintaining epitope accessibility, thereby facilitating effective antigen-antibody interactions. A three-dimensional (3D) model of the MEA was generated using AlphaFold2 (Fig. 1D). The stereochemical quality of the model was then validated by a Ramachandran plot, which showed that 86.7% of residues were located in the most favored regions, 11.4% in additionally allowed regions, and only 1.9% in generously allowed regions (Fig. 1E). This residue distribution indicates a high-quality model with favorable stereochemical properties and structural reliability.

### Expression, purification, and immunoreactivity of the MEA

**Fig 2:**
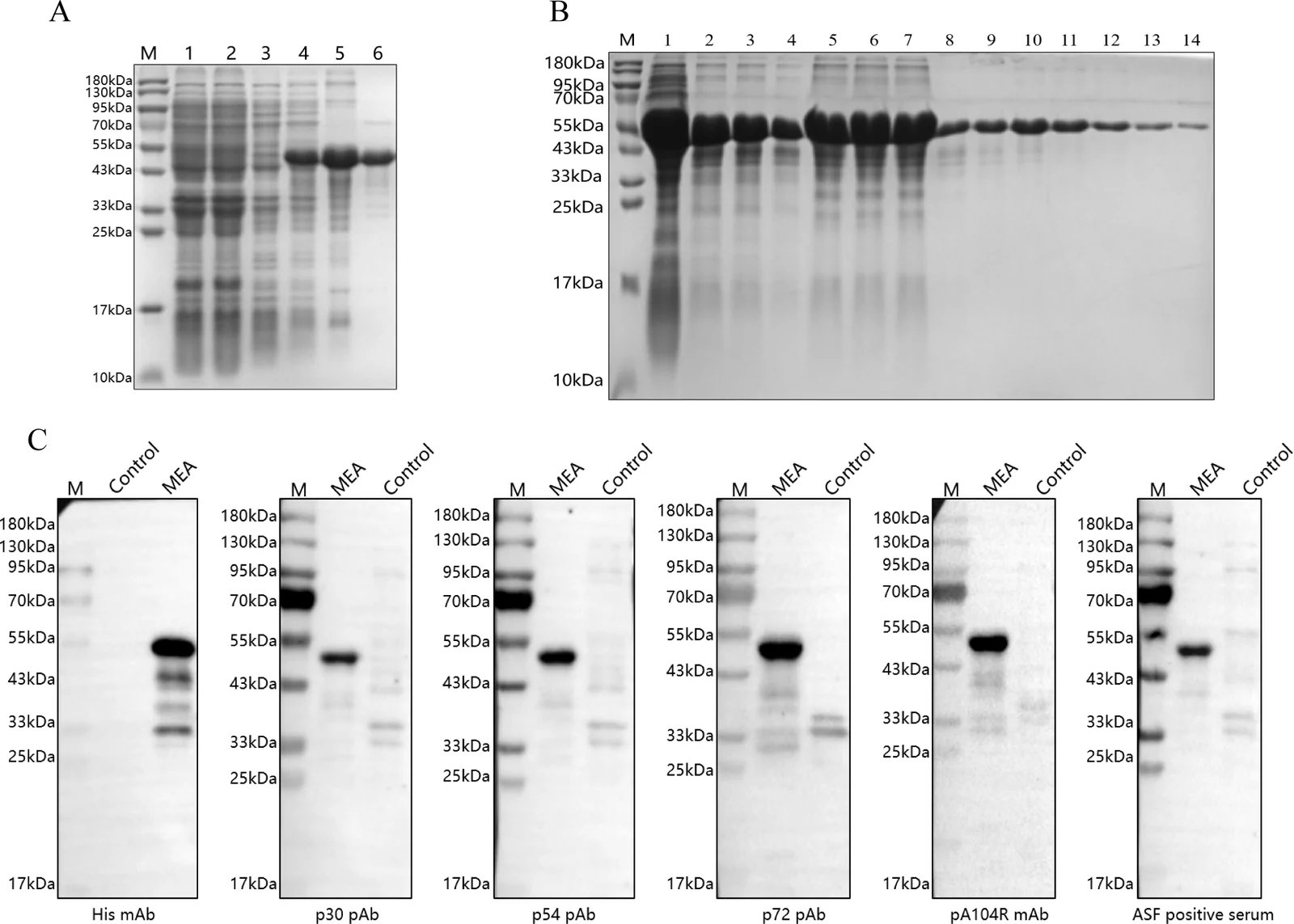
Expression, purification, and identification of the MEA. (**A**) SDS-PAGE analysis of the MEA expression. M, molecular weight markers; lane 1, non-induced vector control bacteria; lane 2, vector bacterial lysate induced for 4 h; lane 3, non-induced MEA-expressing bacterial culture; lane 4, MEA bacterial lysate induced for 4 h; lane 5, insoluble fraction; and lane 6: soluble fraction. (**B**) Purification of His-tagged MEA protein using Ni-NTA affinity chromatography. M, molecular weight markers; lane 1, whole cell lysate; lanes 2–4, wash fractions; lanes 5–7, flow-through fractions; and lanes 8–14, elution fractions collected at imidazole concentrations of 50, 100, 150, 200, 250, 300, and 500 mM, respectively. (**C**) Specificity validation of the purified MEA protein via Western blot. The purified MEA protein and the negative control (lysate of induced bacteria with empty vector) were resolved by SDS-PAGE. The blots were probed with anti-His mAb, anti-p30 pAb, anti-p54 pAb, anti-p72 pAb, anti-pA104R mAb, and ASFV-positive swine serum, respectively.

**Fig 3:**
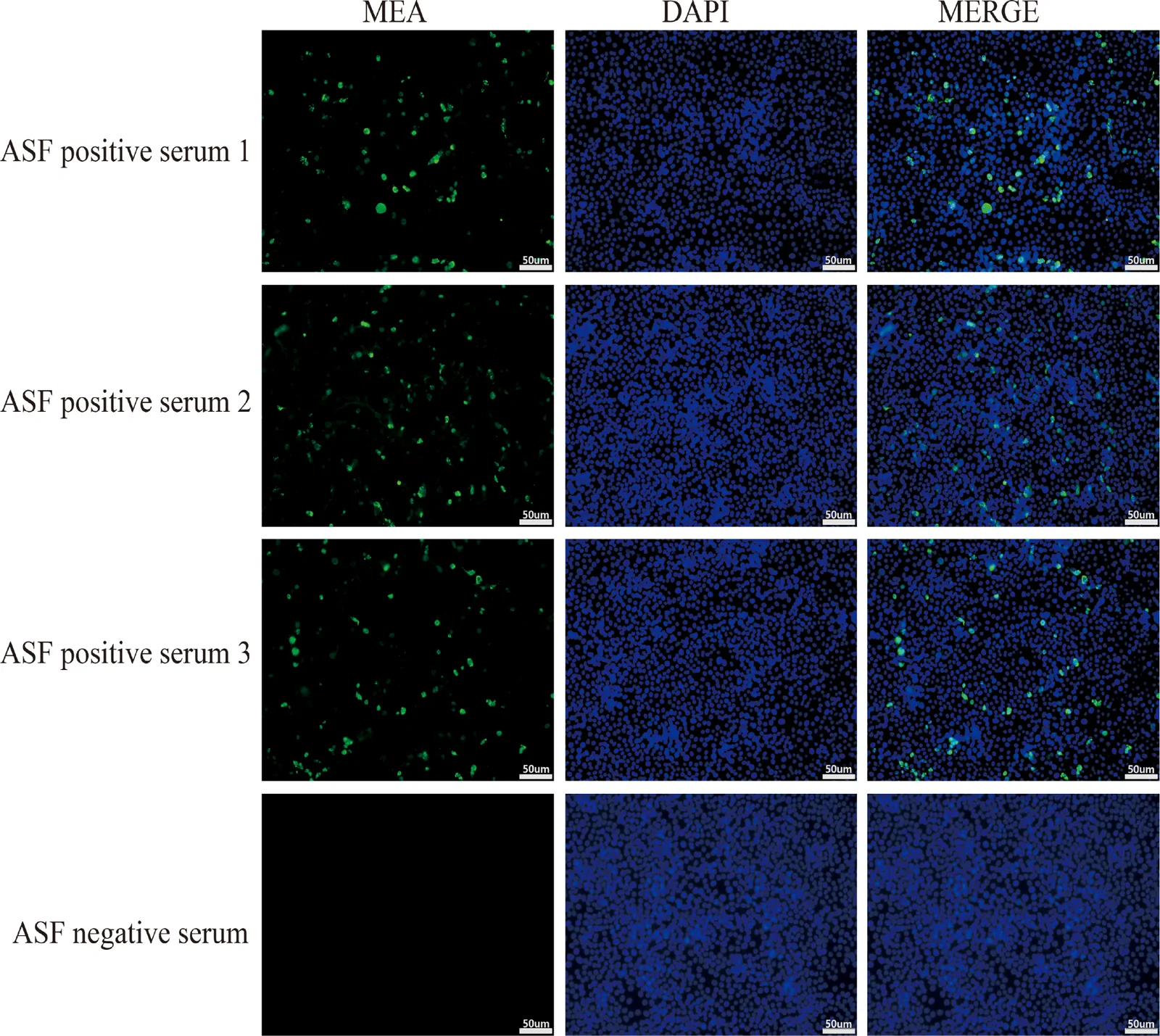
Reactivity of the native MEA protein with naturally derived ASF sera assessed by an indirect immunofluorescence assay. The observed fluorescence demonstrates the binding interaction between the native ASF sera and the native MEA protein. The scale bar in the immunofluorescence image corresponds to 50 µm.

The codon-optimized MEA gene was successfully expressed in *Escherichia coli*. Following induction with isopropyl-β-D-1-thiogalactopyranoside (IPTG), sodium dodecyl-sulfate polyacrylamide gel electrophoresis (SDS-PAGE) analysis of the bacterial lysate revealed a prominent protein band at a molecular weight of ~50 kDa, which was absent in the uninduced control lysate (Fig. 2A). The recombinant His-tagged MEA was then purified from the soluble fraction using Ni-NTA affinity chromatography, yielding a single, highly pure band on SDS-PAGE (Fig. 2B). As shown in Fig. 2C, the immunoreactivity of the purified MEA was confirmed by Western blot analysis. The MEA was specifically recognized by the anti-His-tag antibody, as well as by polyclonal antibodies against p30, p54, p72, a monoclonal antibody against pA104R, and pooled sera from ASFV-infected swine. To further evaluate the immunoreactivity of the MEA, particularly in a folded conformation relevant to ELISA applications, an indirect immunofluorescence assay (IFA) was performed in a eukaryotic system. The MEA expressed in transfected PK-15 cells was strongly recognized by three different ASFV-positive sera, whereas mock-transfected cells exhibited no fluorescence (Fig. 3), confirming its immunoreactivity in a native conformation and supporting its suitability as a diagnostic antigen for serological assays.

### Optimization of the MEA-based indirect ELISA

**Fig 4:**
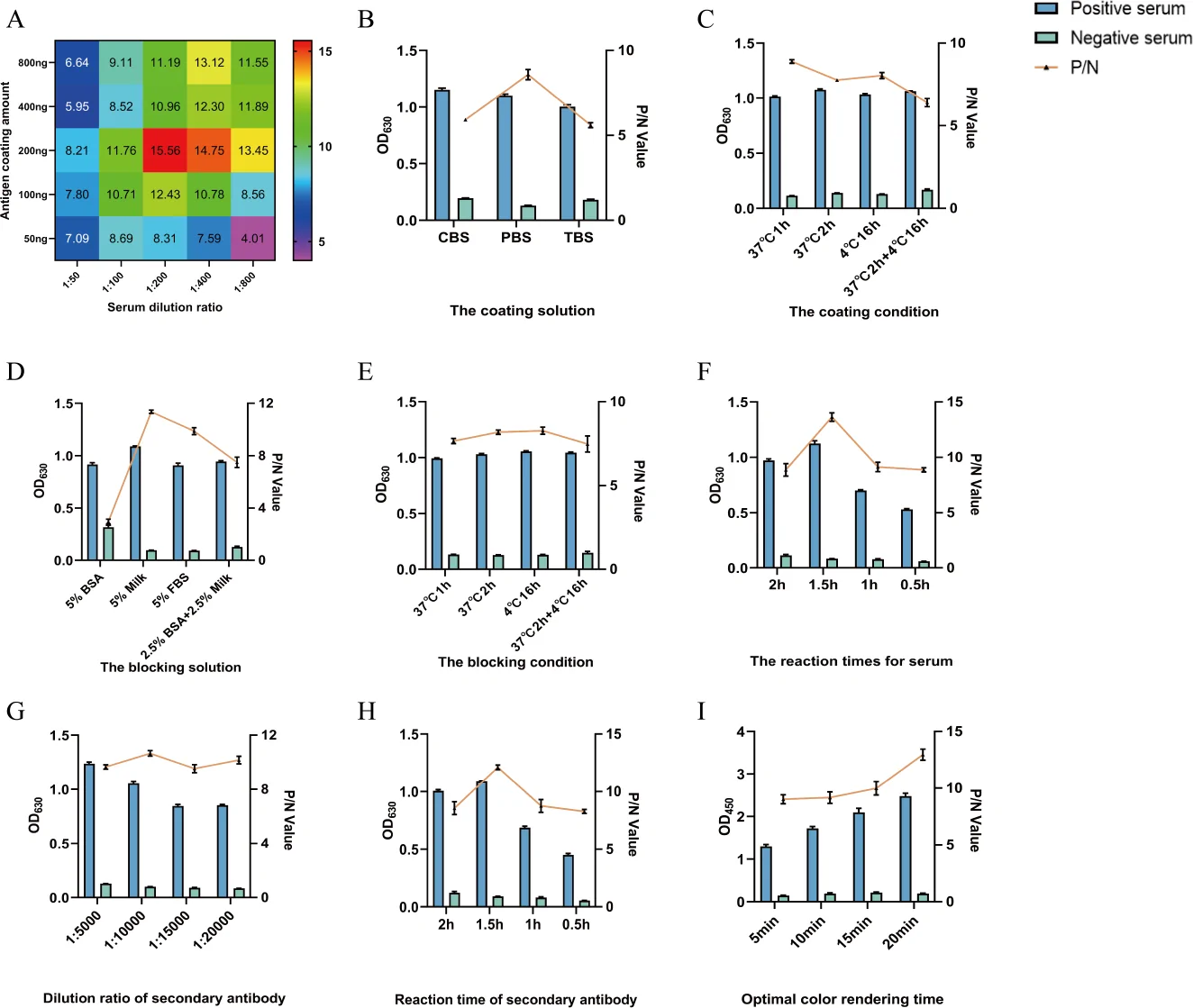
Determination of the reaction conditions, including the optimization of various experimental parameters. (**A**) Screening of the optimal antigen coating amount and serum dilution ratio. (**B**) Evaluation of different coating solutions. (**C**) Assessment of coating conditions. (**D**) Selection of blocking solutions. (**E**) Optimization of blocking conditions. (**F**) Determination of optimal serum incubation conditions. (**G**) Screening of enzyme-labeled antibody dilution ratios. (**H**) Evaluation of enzyme-labeled antibody incubation conditions. (**I**) Analysis of color development conditions at different time points.

To develop a robust iELISA for ASFV antibody detection, a systematic, stepwise optimization of all reaction parameters was performed. The optimal condition for each step was defined as that which yielded the highest positive-to-negative (P/N) ratio while maintaining minimal background signal for negative controls. A checkerboard titration established the optimal antigen coating concentration at 200 ng/well and the optimal serum dilution at 1:200 (Fig. 4A). Based on these optimized parameters, the complete set of optimized conditions was determined as follows: coating with phosphate-buffered saline (PBS) (pH 8.0) at 37°C for 1 h (Fig. 4B and C); blocking with 5% skimmed milk at 37°C for 1 h (Fig. 4D and E); incubating with serum at 37°C for 1.5 h (Fig. 4F); incubating with horseradish peroxidase (HRP)-conjugated secondary antibody at a 1:10,000 dilution for 1.5 h at 37°C (Fig. 4G and H); and developing the colorimetric reaction with TMB substrate for 15 minutes at 37°C (Fig. 4I).

### Determination of cutoff value, specificity, sensitivity, and repeatability

**Fig 5:**
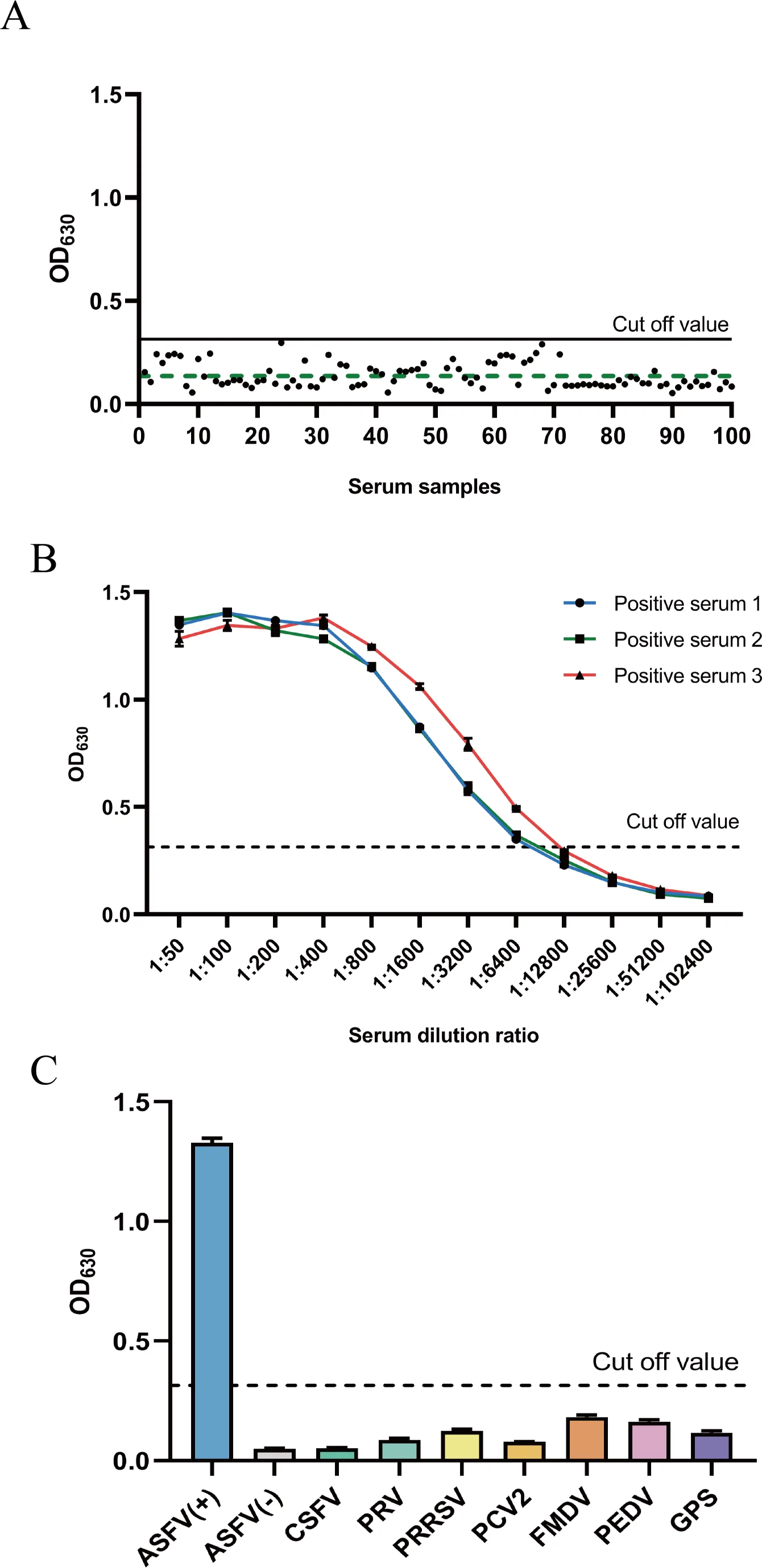
Determination of the cutoff value, sensitivity, and specificity of the iELISA. (**A**) The cutoff value for the iELISA was established at 0.315. (**B**) Assessment of the sensitivity of the iELISA method. The maximum serum dilution yielding an OD_630_ value exceeding the cutoff of 0.315 was determined to be 1:6,400. (**C**) Evaluation of the specificity of the iELISA method, using positive and negative ASFV serum samples as controls.

The diagnostic cutoff value for the iELISA was established based on the analysis of 100 known ASFV-negative serum samples. These samples yielded a mean OD_630_ value of 0.135 with a standard deviation (SD) of 0.060. Therefore, the cutoff value was calculated to be 0.315 (Fig. 5A). Samples yielding an OD_630_ value ≥ 0.315 were considered positive.

The analytical sensitivity was determined by testing serial dilutions of a high-titer ASFV-positive serum. The limit of detection was found to be a dilution of 1:6,400, which still yielded an OD_630_ value above the cutoff, demonstrating the high sensitivity of the assay (Fig. 5B).

To assess specificity, the iELISA was evaluated by testing sera positive for seven other common swine pathogens: classical swine fever virus (CSFV), pseudorabies virus (PRV), porcine reproductive and respiratory syndrome virus (PRRSV), porcine circovirus (PCV), foot-and-mouth disease virus (FMDV), porcine epidemic diarrhea virus (PEDV), and *Glaesserella parasuis* (GPS). As shown in Fig. 5C, no cross-reactivity was observed with any of these sera, indicating the high specificity of the MEA for ASFV antibodies.

The repeatability of the iELISA was assessed by calculating the intra- and inter-assay coefficients of variation (CVs). The intra-assay CVs ranged from 1.75% to 5.08% (Table 4), while the inter-assay CVs ranged from 1.75% to 4.22% (Table 5). Since all CVs were significantly below the 10% threshold, the iELISA was confirmed to have excellent analytical precision and high batch-to-batch reproducibility.

**TABLE 4:** The intra-assay variabilities of the MEA-based iELISA

Serum number	ELISA plate batch (OD_630_ values)						Mean	Standard deviation (SD)	Coefficient of variation (CV%)
	A2	B2	C2	D2	E2	F2			
P5	1.314	1.257	1.263	1.283	1.257	1.264	1.273	0.0222	1.75
P6	1.240	1.199	1.235	1.245	1.262	1.198	1.230	0.0259	2.11
P7	1.034	1.068	1.128	1.098	1.062	1.015	1.068	0.0412	3.86
P8	1.412	1.438	1.452	1.398	1.405	1.456	1.427	0.0250	1.76
N5	0.142	0.153	0.151	0.156	0.143	0.162	0.151	0.0077	5.08
N6	0.101	0.102	0.102	0.103	0.106	0.105	0.103	0.0019	1.88
N7	0.162	0.152	0.158	0.164	0.159	0.163	0.160	0.0044	2.76
N8	0.153	0.158	0.169	0.149	0.163	0.158	0.158	0.0071	4.48

**TABLE 5:** The inter-assay variabilities of the MEA-based iELISA method

Serum number	ELISA plate batch (OD_630_ values)						Mean	Standard deviation	Coefficient of variation (CV%)
	A1	A2	A3	A4	A5	A6			
P1	1.204	1.168	1.210	1.250	1.191	1.218	1.207	0.0274	2.27
P2	1.170	1.175	1.198	1.119	1.239	1.204	1.184	0.0403	3.40
P3	1.133	1.166	1.120	1.124	1.143	1.097	1.131	0.0232	2.05
P4	1.314	1.257	1.263	1.283	1.257	1.264	1.273	0.0222	1.75
N1	0.154	0.155	0.161	0.163	0.154	0.171	0.160	0.0067	4.22
N2	0.104	0.100	0.101	0.102	0.102	0.105	0.102	0.0019	1.82
N3	0.152	0.158	0.164	0.153	0.151	0.156	0.156	0.0048	3.11
N4	0.157	0.161	0.158	0.170	0.162	0.164	0.162	0.0047	2.90

### Diagnostic performance on clinical samples

To assess its diagnostic performance, the established MEA-iELISA was used to test a total of 140 clinical serum samples. The results were compared to those from two commercial kits: the INGEZIM PPA COMPAC (INGENASA) and the ASFV Antibody ELISA Kit (Keqian Biology) (Table 6). The MEA-iELISA achieved the highest positive detection rate of 48.57% (68/140), outperforming the INGENASA kit (44.29%, 62/140) and the Keqian kit (41.43%, 58/140).

**TABLE 6:** Comparison of the MEA-based iELISA with the commercial kits

	MEA-iELISA	INGEZIM PPA COMPAC (INGENASA)	ASFV Antibody ELISA Kit (Keqian Biology)
Positive	68	62	58
Total	140	140	140
Rate (%)	48.57	44.29	41.43

Due to the discrepancies observed, a subset of 45 samples with inconsistent results was further analyzed by ASFV qRT-PCR to establish a reference diagnosis (Table 7). Among these discordant samples, 31 were confirmed to be ASFV-positive by qRT-PCR. The MEA-iELISA correctly identified 25 of these 31 positive samples, demonstrating high diagnostic sensitivity. In contrast, the INGENASA and Keqian kits missed some samples, detecting only 17 and 20, respectively.

**TABLE 7:** Summary of data from three ELISA kits and qPCR determination

Sample	iELISA	INGEZIM PPA COMPAC	ASFV Antibody ELISA Kit		FAM (CT)	HEX (internal control)
O1	+	−		+	27.74	30.68
T44	+	+		−	33.6	30.72
I1	+	−		+	21.92	32.91
I2	+	−		+	28.69	31.04
I3	−	−		+	40+	32.67
I4	−	+		−	40+	30.81
I5	+	−		+	40+	30.73
V2	+	+		−	33.13	30.59
V3	−	+		+	34.67	30.67
V6	+	−		+	30.8	30.79
R3	+	−		+	32.27	30.8
R4	+	+		−	33.89	30.73
R5	−	+		−	40+	32.05
R6	−	−		+	40+	30.93
R16	−	+		+	34.48	30.63
P3	+	−		+	40+	30.83
P8	−	−		+	40+	30.77
P18	+	−		+	29.49	30.85
L13	−	+		−	40+	30.74
L16	+	−		+	27.47	30.6
L113	−	−		+	40+	30.78
R19	+	−		+	33.94	30.77
R20	−	+		+	33.94	31.08
K2	+	+		−	32.74	30.98
K3	+	−		+	33.46	30.77
K4	+	+		−	28.88	30.74
18.67	+	+		−	22.39	32.38
17.96	+	−		+	21.67	32.44
18.51	−	+		+	21.35	33.12
18.84	+	−		+	21.73	32.04
F6-5	+	+		−	32.35	31.42
F6-10	−	+		−	40+	31.83
F7-14	+	−		+	29.5	31.31
11	−	+		+	33.39	30.93
12	+	−		+	34.78	30.84
6	+	+		−	32.14	30.94
2	−	+		−	40+	32.11
7	+	+		−	33.86	31.42
15	−	+		+	32.19	31.2
13	+	+		−	26.77	31.17
3	−	−		+	40+	31.52
8	+	−		+	31.02	32.05
17	−	+		−	40+	33.14
14	+	+		−	21.08	31.02
4	−	+		−	40+	30.98

Regarding the 14 qPCR-negative samples, the MEA-iELISA identified two positive cases. Notably, these positive detections were consistent with the Keqian kit but negative in the INGENASA kit. Collectively, these comparative data demonstrate that the MEA-iELISA possesses enhanced diagnostic sensitivity for detecting ASFV infection, particularly in the early stage of infection, when compared to the two commercial kits evaluated in this study.

## DISCUSSION

ASF continues to pose a significant threat to the global swine industry. In the continued absence of a universally effective vaccine, the prevention and control of this devastating disease remain critically dependent on rapid and accurate diagnostic tools. While molecular assays like qPCR are invaluable for detecting acute infections, serological methods such as ELISA are indispensable for large-scale epidemiological surveillance, the identification of subclinical or chronic carriers, and the detection of infections caused by low-virulence ASFV strains (23). The robust and long-lasting, albeit non-neutralizing, antibody response elicited by ASFV infection solidifies the status of serology as a cornerstone of comprehensive control strategies (24).

The performance of any ELISA is fundamentally determined by the quality and design of its coating antigen (4, 25). Conventional approaches utilize single or multiple full-length recombinant proteins, most commonly p30 (26), p54 (7), the highly conserved p72 (9), and CD2v proteins (27). The combined use of p30 and p54 proteins in serological diagnosis has enhanced the sensitivity for detecting non-parental strains of ASFV (28). In comparison with the IDvet commercial kit, which incorporates three proteins (p30, p72, and pp62), the iELISA method utilizing a recombination of four antigenic proteins (CD2v, CAP80, p54, and p22) has shown superior sensitivity in the early detection of antibodies in pigs infected with ASFV Genotype II (17). While combining multiple proteins can enhance diagnostic sensitivity by capturing a wider array of antibody responses, this approach is fraught with considerable challenges, including high production costs, batch-to-batch variability in protein quality, and difficulties in optimizing the coating ratio of individual antigens. To circumvent these limitations, this study embraced a more rational design strategy by developing a single chimeric MEA. This epitope-based engineering approach not only streamlines production and reduces costs but also allows for the precise arrangement of highly immunodominant epitopes from key ASFV proteins: p30, p54, p72, and pA104R into a single, optimized molecule. These proteins are classical targets for detecting ASFV antibodies, and all have been validated as highly antigenic during infection (29, 30).

A key strength of our study lies in the validation of our *in silico* epitope prediction pipeline. Several B-cell epitopes predicted by our computational analysis for p30, p54, and p72 exhibited remarkable concordance with epitopes that have been experimentally validated in previous studies. Compared to scaffold-based designs (31) or larger 11-antigen constructs (19), our MEA employs a direct tandem fusion of four key immunogens. This results in a more compact (50 kDa) and easily produced antigen that includes pA104R for expanded late-stage detection. In this study, we predicted and selected four epitopes of p30, four of p54, and six of p72 for the design of a multi-epitope recombinant protein. Notably, the predicted epitopes of p30 (Table 1) at positions 68–84 were largely consistent with the experimentally confirmed epitopes 61–90 reported in previous research (32). For p54, the predicted epitope at positions 140–159 matched with epitopes 143–152, as demonstrated in earlier studies (7). Furthermore, the predicted p72 epitopes at positions 31–52 and 185–204 showed a high degree of similarity to the validated epitopes 31–40, 41–45, and 185–189 (10, 33). This alignment between predicted and experimentally validated epitopes provides strong evidence for the reliability of modern immunoinformatic tools and further validates our selection process. Moreover, the subsequent successful expression of the MEA as a stable, soluble protein in *E. coli* further demonstrates the feasibility of translating a computationally designed antigen into a functional biological reagent. This seamless transition from *in silico* design to a tangible protein highlights the efficacy of epitope-based engineering. Notably, the choice of expression system is pivotal. While eukaryotic systems facilitate native folding, they are costly. In contrast, the *E. coli* system circumvents epitope masking potentially caused by extraneous glycosylation, ensuring maximal exposure of B-cell epitopes. Our validation confirms that the prokaryotic-expressed MEA retains high antigenicity and structural integrity, rendering it a cost-effective and practical choice for mass screening.

Beyond epitope selection, an optimized structural design of the MEA was essential for ensuring broad recognition by antibodies. By constructing a design characterized by a high proportion of random coils (52%) and a relatively low content of rigid secondary structures such as alpha-helices and beta-sheets, we sought to enhance the surface exposure and flexibility of the embedded epitopes. This approach is grounded in the established principle that B-cell epitopes are predominantly found in flexible and accessible regions of proteins (34). The strong immunoreactivity of the MEA, confirmed in both its denatured state by Western blot and its native-like conformation by IFA, supports the validity of this design strategy. Consequently, this rational structural design likely contributed to the superior performance of the iELISA, which exhibited high sensitivity, excellent specificity, and consistent reproducibility.

The clinical utility of a new diagnostic assay is best demonstrated through comparative analysis with established methods. When benchmarked against 140 clinical samples, our MEA-iELISA demonstrated a higher positive detection rate than two leading commercial kits. Specifically, in 31 qPCR-confirmed positive samples, the MEA-iELISA detected 25 cases, surpassing the INGENASA kit (17/31) and the Keqian kit (20/31). This augmented sensitivity is likely because the INGENASA kit relies on a single p72 antigen, while the Keqian kit, which utilizes whole-protein antigens, may suffer from steric hindrance or epitope masking. Furthermore, for qPCR-negative/ELISA-positive samples, the consistency between the MEA-iELISA and the Keqian kit suggests the presence of convalescent animals, although the possibility of non-specific binding cannot be entirely ruled out without definitive infection histories.

Finally, the potential influence of ASFV immunological diversity should be considered. While pA104R and p72 are highly conserved (>97% similarity), p30 and p54 show significant differences (84%–86%) among different strains. To address this, our *in silico* design accounted for sequence conservation across Genotype I and Genotype I/II hybrids to maximize potential cross-reactivity. Although optimized for Genotype II, the assay’s performance against other strains, such as Genotype I or I/II hybrids, remains to be fully characterized. Furthermore, the few missed qPCR-positive samples suggest that individual variability in immune responses to specific linear epitopes can lead to occasional false negatives. Future studies with more genotypes will help evaluate the broad effectiveness of this tool. This study, therefore, not only presents a new diagnostic tool but also highlights a powerful and adaptable methodology for developing next-generation serological assays for ASF and other infectious diseases.

### Conclusion

In this study, we successfully employed a rational, immunoinformatics-driven approach to design, express, and validate a novel multi-epitope recombinant antigen, MEA, incorporating key B-cell epitopes from four major ASFV structural proteins. The iELISA developed using this MEA as the coating antigen demonstrated high sensitivity, specificity, and repeatability for the detection of ASFV antibodies in swine serum. Notably, comparative evaluation with clinical samples revealed that the MEA-iELISA exhibits superior diagnostic sensitivity over commercial kits, particularly in detecting early-stage infections. Collectively, this work establishes the MEA-iELISA as a highly effective and potentially cost-efficient tool for clinical diagnosis and large-scale serological surveillance, whose implementation could significantly contribute to the ongoing global effort to control and eradicate ASF.

## MATERIALS AND METHODS

### Cells, reagents, and antibodies

*Escherichia coli* strains DH5α and BL21 (DE3) (Tsingke Biotechnology, Wuhan, China) were used for plasmid propagation and recombinant protein expression, respectively. The gene encoding the multi-epitope antigen was codon-optimized for expression in *E. coli* and synthesized by GenScript (Nanjing, China). Porcine kidney (PK-15) cells were cultured in Dulbecco’s modified Eagle’s medium supplemented with 10% fetal bovine serum (FBS).

The primary antibodies included a monoclonal anti-His-tag antibody (Abmart, Shanghai, China) for Western blot analysis. Monospecific polyclonal antibodies against ASFV p30, p54, and p72, as well as a monoclonal antibody against pA104R, were prepared and stored in our laboratory. A panel of characterized ASFV-positive and negative swine sera was maintained in our laboratory (35). Secondary antibodies, including horseradish peroxidase-conjugated goat anti-mouse IgG, HRP-conjugated rabbit anti-pig IgG (Biodragon, Beijing, China), and FITC-conjugated rabbit anti-porcine IgG (Proteintech, Chicago, USA) were used for Western blotting and IFAs, respectively.

### Epitope prediction

The amino acid sequences of p30, p54, and p72 proteins from the ASFV-SY18 strain (GenBank: MH766894.2) were retrieved from the National Center for Biotechnology Information. B-cell epitopes within these proteins were predicted using the Immune Epitope Database and SVMTriP bioinformatics tools, while the prediction threshold for both IEDB and SVMTriP was set to 0.5. All protein sequences were analyzed in FASTA format.

### Design and *in Silico* analysis of the recombinant multi-epitope antigen

The final MEA construct was rationally designed to incorporate 15 B-cell epitopes. Fourteen high-scoring epitopes were selected from p30, p54, and p72 proteins based on the aforementioned predictions, while one epitope from the pA104R protein was included based on our previous research (36). These 15 epitopes were genetically fused in tandem using a combination of flexible “GGGS” and rigid “KK” linkers. The antigenicity of the MEA was evaluated using the VaxiJen v.2.0 server (with a threshold of 0.4) and the ANTIGENpro server (with a threshold of 0.5), while potential allergenicity was assessed using AllerTop and AllergenFP. Physicochemical properties, including theoretical isoelectric point (pI), instability index, aliphatic index, and grand average of hydropathicity, were calculated using ExPASy ProtParam.

The secondary structure was analyzed using the DNASTAR Protean and PSIPRED v.4.0. The 3D structure was modeled using the AlphaFold2 (ColabFold), and its stereochemical quality and structural validity of the final 3D model were assessed via Ramachandran plot analysis using the PROCHECK web server.

### Expression and purification of the recombinant antigen

The recombinant plasmid pET-30a(+)-MEA was transformed into *E. coli* BL21 (DE3)-competent cells. A single colony was cultured into Luria-Bertani broth containing 50 µg/mL kanamycin and grown at 37°C with shaking. When the OD_600_ reached 0.6–0.8, MEA expression was induced with 0.6 mM IPTG (Solarbio, Beijing, China) for 4 h at 37°C. Cells were harvested by centrifugation, resuspended in PBS, and lysed using an ultrahigh-pressure cell disrupter. The expression of the recombinant protein was confirmed by sodium dodecyl-sulfate polyacrylamide gel electrophoresis. The His-tagged MEA was purified from the soluble fraction using a Ni-NTA affinity column (Cytiva, CA, USA) according to the manufacturer’s protocol. The concentration of the purified protein was determined using a BCA protein assay kit (Beyotime, Shanghai, China).

For Western blot analysis, the purified protein was separated by SDS-PAGE and transferred to a polyvinylidene difluoride membrane (Millipore, Darmstadt, Germany). The membrane was blocked with 5% bovine serum albumin (BSA) in Tris-buffered saline with 0.1% Tween-20 for 2 h at room temperature. Subsequently, membrane strips were incubated overnight at 4°C with primary antibodies. After washing, the membrane was incubated with the corresponding HRP-conjugated secondary antibody for 1 h. Protein bands were visualized using the ChemiDoc XRS1 imaging system (Bio-Rad Laboratories, Hercules, USA).

### Indirect immunofluorescence assay

PK-15 cells were seeded onto glass coverslips in a 12-well plate. Upon reaching 80% confluency, cells were transfected with the pcDNA3.1(+)-MEA plasmid. At 24 h post-transfection, cells were fixed with 4% paraformaldehyde for 20 minutes, permeabilized with 0.1% Triton X-100 in PBS, and blocked with 5% BSA in PBS for 1 h at 37°C. The cells were then incubated with ASFV-positive swine serum (1:200 dilution) overnight at 4°C, followed by incubation with FITC-conjugated rabbit anti-porcine IgG (1:500 dilution) for 1 h at 37°C. Nuclei were stained with DAPI (Beyotime, Shanghai, China). Finally, the coverslips were mounted using an antifade mounting medium (Beyotime, Shanghai, China) and imaged with a fluorescence microscope (EVOS FL Auto, Thermo Scientific, USA).

### Establishment and optimization of the indirect ELISA

To establish an iELISA using the purified MEA, a systematic, one-variable-at-a-time optimization of all reaction parameters was performed to achieve the highest signal-to-noise ratio. Initially, a checkerboard titration determined the optimal antigen coating concentration (ranging from 50 to 800 ng/well) and the ideal primary serum dilution (from 1:200 to 1:3,200). Subsequently, various coating conditions were evaluated, including coating buffers (0.01 M carbonate buffer, pH 9.6; 0.01 M PBS, pH 8.0; and 0.05 M Tris-HCl, pH 9.6) and four incubation protocols (37°C for 1 h, 37°C for 2 h, 37°C for 2 h followed by 4°C for 16 h, and 4°C for 16 h). The blocking step was compared using 5% BSA, 5% FBS, 5% skimmed milk, or a BSA/skimmed milk combination in PBST. Further optimization determined the ideal incubation times for serum and HRP-conjugated secondary antibody (0.5, 1, 1.5, and 2 h), the optimal secondary antibody dilution (1:5,000, 1:10,000, 1:15,000, and 1:20,000), and the TMB substrate reaction time (5, 10, 15, and 20 minutes) at 37°C. All optimization experiments were performed in triplicate, and the condition yielding the highest positive-to-negative (P/N) ratio was selected for the finalized assay protocol.

### Determination of cutoff value, specificity, sensitivity, and repeatability

The diagnostic cutoff value was established by testing 100 ASFV-negative sera and was calculated as the mean optical density at 630 nm plus three standard deviations (x̄ + 3 SD). Analytical specificity was assessed by testing a panel of porcine serum samples positive for other major swine pathogens, including CSFV, PRV, PRRSV, PCV2, FMDV, PEDV, and GPS.

Analytical sensitivity (limit of detection) was determined by testing serial twofold dilutions (from 1:50 to 1:102,400) of a high-titer ASFV-positive serum. The intra- and inter-assay coefficients of variation were calculated to evaluate assay precision. For the intra-assay CV, eight serum samples (four positive and four negative) were tested in six replicates on the same plate. For the inter-assay CV, the same eight samples were tested across six different occasions.

### Comparison with commercial ELISA kits

To evaluate diagnostic performance, the MEA-iELISA was benchmarked against two commercially available kits: a p72 antigen-based ELISA (INGEZIM PPA COMPAC; INGENASA, Madrid, Spain) and a multi-antigen ELISA (p30, p54, and p72-based, ASFV Antibody ELISA Kit, Keqian Biology, Wuhan, China). A panel of clinical swine serum samples was tested in parallel. The presumed true infection status of these samples was independently confirmed by a commercial ASFV qRT-PCR kit (Keqian Biology, Wuhan, China), which served as the reference standard. The relative sensitivity and concordance between the in-house iELISA relative to the reference standard and the commercial kits were then calculated.

### Statistical analysis

All data were analyzed using GraphPad Prism 9.5.0. The cutoff value and intra- and inter-assay CVs were calculated to evaluate the repeatability and reproducibility of the assay, respectively.

## Data Availability

The data sets used during the current study are available from the corresponding author upon reasonable request.
